# Prevalence and clinical characterization of oral clefts in patients with chromosome trisomy 18

**DOI:** 10.1590/1984-0462/2024/42/2023169

**Published:** 2024-06-24

**Authors:** Nadini Cristina Marins Martinez, Sheila Tamanini de Almeida, Rafael Fabiano Machado Rosa, Paulo Ricardo Gazzola Zen

**Affiliations:** a Universidade Federal de Ciências da Saúde de Porto Alegre – Porto Alegre, RS, Brazil.; b Irmandade da Santa Casa de Misericórdia de Porto Alegre – Porto Alegre, RS, Brazil.

**Keywords:** Edwards syndrome, Trisomy 18, Cleft palate, Cleft lip, Genetics, Síndrome de Edwards, Trissomia 18, Fenda palatina, Fenda labial, Genética

## Abstract

**Objective:**

To verify the prevalence and perform the clinical characterization of oral clefts in a sample of patients with trisomy of chromosome 18 in Southern Brazil.

**Methods:**

This was a retrospective cross-sectional study, performed in a reference clinical genetic service in Southern Brazil. The initial sample consisted of 77 patients diagnosed in the neonatal period with trisomy 18 treated at the Clinical Genetics Service of a referral hospital at Federal University of Health Sciences of Porto Alegre (UFCSPA). The patients’ diagnosis was confirmed by karyotype and care was provided during their stay in the intensive care unit (ICU) of the hospital that is a reference in Southern Brazil for care for malformed patients. The period covered was from 1975 to 2020.

**Results:**

During the study period, 77 patients diagnosed with trisomy 18 were treated, most of them in the ICU. Of these, 13 individuals were excluded due to incomplete data. The final sample consisted of 64 patients with an average age of 2.4 years of life, ranging from one day to 16 years old, the majority of whom were female. Regarding face dysmorphisms identified in the sample, three (4,68%) patients had cleft lip and two (3,11%) had cleft lip and palate.

**Conclusions:**

This study contributed to the recognition of the characteristics and prevalence of oral clefts in individuals with trisomy 18 in a sample of patients from Southern Brazil. In addition, we described the clinical alterations found in patients with oral clefts, as well as other associated comorbidities, such as cardiac, neurological and pulmonary comorbidities, as well as cranial and facial dysmorphisms.

## RESUMO

**Objetivo:**

Verificar a prevalência e realizar a caracterização clínica das fissuras orais em uma amostra de pacientes com trissomia do cromossomo 18 no sul do Brasil.

**Métodos:**

Este foi um estudo transversal retrospectivo, realizado em um serviço de referência em genética clínica do sul do Brasil. A amostra inicial foi composta de 77 pacientes com diagnóstico no período neonatal de trissomia 18 atendidos no Serviço de Genética Clínica da Universidade Federal de Ciências da Saúde de Porto Alegre (UFCSPA). O diagnóstico dos pacientes foi confirmado por cariótipo e os atendimentos foram realizados durante sua internação na unidade de terapia intensiva (UTI) de hospital de referência no sul do Brasil para atendimento em pacientes malformados. O período abrangido foi de 1975 a 2020.

**Resultados:**

Durante o período do estudo foram atendidos, a maioria na UTI do hospital, 77 pacientes com diagnóstico de trissomia do cromossomo 18. Destes, 13 indivíduos foram excluídos por apresentarem dados incompletos. A amostra final foi de 64 pacientes, com idade média de 2,4 anos de vida, variando de um dia de vida a 16 anos, a maioria do sexo feminino. Com relação aos dismorfismos faciais identificados na amostra, três (4,68%) pacientes apresentavam fissuras labiais e dois (3,11%) fissuras labiopalatinas.

**Conclusões:**

Este estudo trouxe como contribuições o reconhecimento das características e a prevalência das fendas orais nos indivíduos com trissomia do cromossomo 18 em uma amostra de pacientes do sul do Brasil. Além disso, descrevemos as alterações clínicas encontradas em pacientes com fissuras orais, bem como outras comorbidades associadas, como comorbidades cardíacas, neurológicas e pulmonares, além de dismorfismos cranianos e faciais.

## INTRODUCTION

Trisomy 18, also known as Edwards syndrome, is a chromosomal abnormality caused by the presence of an extra chromosome 18. The syndrome encompasses a set of major and minor malformations and is associated with high neonatal risk, high infant mortality, and delay in neuropsychomotor development.^1,2^

The most important factor for the occurrence of trisomy 18 is advanced maternal age, which predisposes to non-disjunction of chromosomes in gametogenesis, more specifically in the meiosis II phase.^3,4^ However, there are cases in which this syndrome occurs due to chromosomal translocations, which may be new alterations or transmission over generations, and to chromosomal mosaicism, always being a postzygotic effect.^5,6^

Trisomy 18 has a wide range of clinical manifestations, affecting different body systems, with more than 130 anomalies being described in the literature.^6^ The most common phenotypic characteristics of this syndrome are neurological, growth, skull and face, thorax and abdomen, extremities, genitalia, skin and phanera, and internal organs alterations.^7^

Among the cranial and facial abnormalities, the skull with prominent occipital region, large fontanelles, and microcephaly can be highlighted, besides triangular face, with broad forehead, narrow palpebral fissures, small nose and mouth, ogival palate, micrognathia, ears with low implantation, and cleft lip and palate.^8,9^

Non-syndromic orofacial clefts, which include cleft lip, cleft lip and palate, and cleft palate itself, comprise a range of diseases that affect the lips and oral cavity, the causes of which remain largely unknown.^10,11^ Among non-isolated cases, approximately 50% are multiple congenital anomalies without a specific diagnosis, while the remainder correspond to syndromic cases, with chromosomal, genetic, or teratogenic etiology. Effects on hearing, language, and feeding process, which affects all functions of the stomatognathic system, sucking, chewing, swallowing, phonoarticulation, and breathing, in addition to cognition and appearance can lead to long-term adverse results for health and social integration.^12,13^

Affected children require multidisciplinary care from birth through adulthood and have higher lifetime morbidity and mortality than unaffected individuals.^14,15^

Trisomy 18 generates clinical manifestations in the craniofacial and orofacial regions, and may, therefore, result in oral clefts, which significantly impact an individual’s life. Cleft lip was described in about 5% of cases and cleft palate in another 5% in this group of patients.^15^

In this context, this work aimed to verify the prevalence and perform the clinical characterization of oral clefts in a sample of patients with trisomy 18 of Southern Brazil.

## METHOD

This was a retrospective cross-sectional study. The initial sample consisted of 77 patients diagnosed with trisomy 18 attended by the Clinical Genetics Service of the Federal University of Health Sciences of Porto Alegre (UFCSPA) during their hospitalization in the intensive care unit (ICU) at the hospital *Irmandade da Santa Casa de Misericórdia de Porto Alegre* (ISCMPA) from 1975 to 2020. As ISCMPA is a reference in the care of malformed patients, cases are referred from all over Rio Grande do Sul. The study was approved by the institutional research ethics committee, under protocol 5.446.128. For this study, consent was waived since it is a retrospective study; therefore, data were taken from medical records, with Ethics Commission approval.

For data collection, a review of the patients’ charts was carried out and a standard protocol was completed. The data collected consisted of age at diagnosis, reason for referral, evaluation period, karyotype result, gestational and perinatal histories, birth data, family history, physical examination at the first evaluation, craniofacial abnormalities, and other dysmorphias, complementary exams, surgeries, complications, and outcomes. Dysmorphias were divided according to the affected anatomical region.

## RESULTS

The records of 77 patients diagnosed with Edwards syndrome at the Clinical Genetics Service were reviewed. However, 13 individuals were excluded for presenting incomplete data, thus leaving a sample of 64 patients. The average age was 2.4 years of life, ranging from one day to 16 years old, with 44 (68.75%) females and 20 (31.25%) males.

The mean maternal age was 36.1 years in a range of 15 to 52 years. An important factor to be described in this regard is the high percentage of mothers over 35 years old, as there were 26 (40.62%). Concerning gestational age, 27 (42.18%) were born at less than 37 weeks, therefore, prematurely, and 25 were born at term. These women reported using some medications during the gestational period, such as levothyroxine, methyldopa, scopolamine/dipyrone butylbromide, gentamicin, ampicillin, ferrous sulfate, and fluoxetine. One of them used the abortive medication misoprostol. Among the 64 (83.11%) mothers of patients with Edwards syndrome, four (6.25%) reported tobacco use, two (3.12%) alcohol use, and two (3.11%) used both. Of the cleft patients, only the mother of patient B used tobacco during pregnancy.

Regarding face dysmorphisms identified in the sample, three (4.68%) patients had cleft lip and two (3.11%) had cleft lip and palate. In addition, 40 (62.50%) patients had micrognathia. The data cited above can be seen in Table 1.

**Table 1:** Characterization of patients with oral clefts.

Patient	Sex	Age at assessment date (days)	Reason for requesting the evaluation	Cleft type	Karyotype
PA	F	11	Syndromic aspect, heart disease	Bilateral cleft lip and palate	47,XX,+18
PB	F	1	Syndromic aspect	Cleft lip bilateral	47,XX,+18
PC	M	382	Kidney anomaly (caliceal dilation, with evidence of ureter dilation)	Cleft lip bilateral	46,XY, der(5), t(5;18) (q34;q11.2)
PD	M	8	Assessment as Edwards syndrome suspicion	Cleft lip bilateral	47,XY,+18
PE	F	7	VSD, long bone reduction, ambiguous genitalia	Cleft lip and palate	47,XX,+18

VSD: ventricular septal defect; F: female; M: male.

Among the skull dysmorphias, the most cited were occipital prominence in 28 (43.75%) of patients, dolichocephaly in 11 (17.18%), and occipital encephalocele in two (3.11%). Ear dysmorphias were of low implantation and retroverted in 37 (57.81%) patients, hypoplastic in five (7.81%), and microtia in two (3.11%). As for the abnormalities in the abdomen, there were inguinal hernias in six patients (9.37%) and omphaloceles in three (4.68%). In terms of skin and adnexa abnormalities, our sample showed excess skin in the cervical region and lanugo on the back.

In the nervous system, we verified one presence of hydrocephalus (1.56%), three ventricular dilatation (4.68%), one dysgenesis of the corpus callosum (1.56%), and two posterior pits with aspect of the Dandy-Walker variant or megacistern magna (3.11%).

The most frequent cardiac alterations were interventricular communication (IVC) presented in 23 (35.93%) individuals, and 15 (23.43%) had interatrial communication (atrial septal defect — ASD), besides cardiomegaly, murmur, aortic prolapse, and tricuspid dysplasia. Other abnormalities were described as pulmonary stenosis and hypoplastic pulmonary valve. All of these, therefore, were clinical findings observed in patients with trisomy 18, as shown in Table 2.

**Table 2:** Clinical findings observed in patients with trisomy 18 and oral clefts.

	With oral clefts				
	PA	PB	PC	PD	PE
Age (days)	11		382	8	7
Age at death (days)	-	-	-	18	73
Face dysmorphia	Micrognathia	Micrognathia	Midface hypoplasia	Micrognathia	Hypoplastic nasal bone
Skull dysmorphia	Prominent occiput	+ (ND)	+ (ND)	Prominent and broad forehead, microphthalmia	+ (ND)
Ears	NI	Low implantation, hyperfold helix	NI	Low and retroverted implantation	NI
Skeletal abnormalities	Hypertonia, short chest	Thorax excavatum	+ (ND)	Hypertonia	Hypoplastic middle phalanx, reduction of long bones
Skin abnormalities	Excess skin in the cervical region and lanugo on the back	Hirsutism	NI	NI	NI
Nervous system changes	NI	NI	NI	NI	NI
Congenital heart defects	+ (ND)	PFO, PDA, VSD, aortic regurgitation	NI	Fallot tetralogy, pulmonary valve atresia, mild aortic valve alteration, patent ductus, double right ventricle outlet	Perimembranous VSD and small oval fossa type ASD, dysplastic aortic valve

NI: Not informed; ND: Not described; d: Days of life; PFO: patent foramen ovale; PCA: Persistence of ductus arteriosus; VSD: Ventricular septal defect; ASD: Atrial septal defect. In some old medical records, dysmorphia was mentioned as present but was not detailed

## DISCUSSION

In the present study, we observed a relationship between oral cleft and trisomy 18. Although the sample was small, among the 64 individuals, five (7.81%) had some type of oral cleft. The analysis was descriptive, therefore, without the use of a statistical program due to the sample size as previously reported. In addition, it was not possible to identify the pathophysiological etiological mechanism involved given the multisymptomology of the cases. The frequency of oral clefts in patients with trisomy 18 was like other studies described in the literature. We also identified the frequent presence of other concomitant anomalies.

Perrotin et al.^16^ at the University Hospital of Bretonneau, France, investigated chromosomal defects and associated malformations in 62 fetuses with orofacial clefts. They verified that, of the 62 fetuses with orofacial clefts, five (8.06%) had trisomy 18. The study showed that orofacial clefts in patients with this syndrome are associated with other abnormalities, such as micrognathia, strawberry-shaped head, diaphragmatic hernia congenital, cerebellar hypoplasia, choroid plexus cysts, posterior fossa cyst, intrauterine growth retardation, ventricular septal defect, transposition of vessels, absence of radius, overlapping toes, hydronephrosis, congenital clubfoot, and foot on blotter or chair balance.

In 2006, a study carried out by Chen^17^ investigated 89 patients with trisomy 18 at Mackay Memorial Hospital, Taiwan. Of these, nine (10.11%) had palpebral fissures. The study showed that orofacial clefts were associated with wrist and ankle arthrogryposis (uni or bilateral), radial aplasia (uni or bilateral), ulnar hypoplasia, echogenic bowel, semilobar holoprosencephaly, tracheoesophageal fistula, choroid plexus cysts, intrauterine growth restriction, cloacal dysgenesis, umbilical cord cysts, omphalocele, and megacysts.^18-20^

In the French study by Perrotin et al. mentioned above, among the five fetuses that had trisomy 18, three (60%) had a bilateral cleft.^16^ And, in the Chinese study by Chen also cited above, of the nine cases of orofacial clefts in patients with trisomy 18, all (100%) were cleft lips and palates; five were median and four were bilateral. Therefore, it is possible to notice an association of orofacial clefts in individuals with trisomy 18 with other clinical alterations. The first study that noticed an association between orofacial clefts and choroid plexus cysts was that of Perrotin et al. Chen says that it is common for orofacial clefts to be associated with arthrogryposis and semilobar holoprosencephaly.^16^

Hsiao et al.^20^ investigated a sample of 31 children with trisomy 18 from the Changhua Christian Hospital in Taiwan, China. The clinical characteristics presented by these patients were diverse but none had orofacial clefts. The most prevalent clinical manifestations were uterine growth retardation (90% of cases), congenital heart disease (77%), malformation and poor implantation of the ears (71%), small mouth opening (61%), micrognathia (58%), hand with overlapping toes (58%), occipital prominence (55%), foot on blotter or rocking chair (52%), hypo or hypertonia (52%) and microcephaly (52%). In 2006, Hodes et al.^9^ gathered a sample of 28 patients with trisomy 18 in the United States. As in the aforementioned study, the clinical characteristics presented by these patients were diverse but none presented orofacial clefts. The most prevalent clinical manifestations were ear abnormalities (100%), cardiac abnormalities (93%), micrognathia (86%), hand with overlapping fingers (89%), occipital prominence (70%), microcephaly (65%), and dislocated hip (62%). Both studies corroborate our findings, as we obtained a significant percentage in our sample of patients with micrognathia, as well as congenital heart diseases.

There are several recognized environmental risk factors and several genes involved in the etiology of orofacial clefts, which means that the etiology is complex and heterogeneous. Smoking during pregnancy is a recognized risk factor for orofacial clefts. It is estimated that 6.1% (95% confidence interval [CI] 4.4–7.7%) of orofacial clefts could be prevented by eliminating smoking.^21-24^ In our sample, the mother of patient B had used tobacco during pregnancy.

Maternal age is described as a risk factor for orofacial clefts. In the study by Perrotin et al. it is possible to observe the relevance of advanced maternal age in the individuals of their sample, which also occurs in our study with a prevalence of 40.62% of mothers over 35 years of age. In addition, maternal age is also shown to be a risk factor for trisomy 18. In Edwards syndrome, as in other trisomies, maternal age is increased.^16^

In the literature, there seems to be no doubt that this is the most significant predisposing factor for the non-disjunction of chromosomes during cell division. Most cases of trisomy 18 occur due to de novo meiotic non-disjunction in maternal meiosis II. ^25,26^

Other evidence is about the consumption of alcohol by the mother as a risk factor, however less consistent. It is known that even if the pregnant woman consumes large amounts in a short period, it can increase the risk of cleft.^27-30^ Socioeconomic aspects have been suggested as risk factors but it is difficult to separate the combined effects of nutrition and maternal health. Several prescription drugs also increase risk when taken during the first trimester, including folate antagonists and some other drugs that exhibit anti-folate properties.^31,32^

It is important to highlight that most of the studies found do not clearly specify the type of cleft present, only the registration and also the relationship with the population studied, trisomy 18, in this case. Furthermore, we described the clinical changes found in patients with oral clefts, as well as other associated comorbidities, such as cardiac, neurological, and pulmonary, in addition to cranial and facial dysmorphisms. As far as we know, this work is one of the few in Brazil and Latin America contrasting oral clefts and trisomy 18.

The main limitation of the present study is the fact that it is retrospective: many medical records were lost, and many had incomplete data. Therefore, only the cases in which we had more information in the medical records remained, providing the research with a more well-founded basis, and avoiding possible biases as much as possible. This care had a positive impact on the organization and presentation of results. However, the reduction of the sample size increased the chance of beta errors.

It is fundamental to emphasize that among our patients diagnosed with trisomy 18, five cases presented, among many other deformities, some type of oral cleft. Therefore, we can consider it relevant, since they represent 7.81% of our sample. These patients will require additional care related to the presence of fissures, including care from a multidisciplinary team that can provide the necessary support according to each patient and his/her individuality, as they directly impact the morbidity and mortality of this group.

This study contributed to the recognition of the characteristics and prevalence of oral clefts in individuals with trisomy 18 in a sample of patients from Southern Brazil. Furthermore, we described the clinical changes found in patients with oral clefts, as well as other associated comorbidities, such as cardiac, neurological, and pulmonary, as well as skull and facial dysmorphia.

## Data Availability

The database that originated the article is available with the corresponding author.
